# Organizational support and task performance: a multiple mediation model

**DOI:** 10.3389/fpsyg.2023.1258177

**Published:** 2024-01-04

**Authors:** Xiaoyuan Chu, Jingyue Yu, Alafate Litifu, Wenlu Zhao, Xinyi Wei, Pengcheng Wang, Jun Wei

**Affiliations:** ^1^School of Economics and Management, Beijing University of Posts and Telecommunications, Beijing, China; ^2^School of Network Education, Beijing University of Posts and Telecommunications, Beijing, China; ^3^Department of Psychology, Renmin University of China, Beijing, China; ^4^School of Media and Communication, Shanghai Jiao Tong University, Shanghai, China; ^5^School of Business, NingboTech University, Ningbo, China

**Keywords:** organizational support, task performance, organizational commitment, job satisfaction, auditors

## Abstract

**Objective:**

Organizational support has been identified as one of the causes for task performance, while previous studies have not adequately explored the underlying mechanisms. Thus, this study aims to reveal the potential mechanisms that linked organizational support to task performance.

**Methods:**

A questionnaire survey was conducted among the 720 participants from 12 audit firms in Beijing in December 2020. Participants completed anonymous questionnaires assessing their organizational support, task performance, organizational commitment, and job satisfaction. Data analysis was conducted with SPSS 26.0.

**Results:**

(1) Organizational support was positively associated with task performance, (2) job satisfaction and organizational commitment both mediated the relationship between organizational support and task performance respectively, and (3) the relationship between organizational support and task performance was also serially mediated by job satisfaction and then organizational commitment.

**Conclusion:**

Job satisfaction and organizational commitment played a serial multiple mediating role in the association between organizational support and task performance, which provides a potential path for improving task performance.

## 1 Introduction

Task performance can be understood as behavioral patterns that are directly involved in production and service provision, or activities that offer support indirectly to the organization’s core technical processes (Van Scotter et al., 2000). It encompasses activities prescribed by the formal job role (Shen and Benson, 2016), which is related to the execution and maintenance of core technical processes in an organization (Motowidlo et al., 1997). It reflects an employee’s effectiveness in completing the core job or role-based responsibilities (Singh, 2019). Empirical studies suggest that task performance not only facilitates employees’ professional advancement (Van Scotter et al., 2000) but also promotes accomplishment of organizational goals (Tett and Burnett, 2003). An important potential cause for task performance is organizational support. Organizational support refers to the value that organizations place on the contributions of their members and the concern for their well-being (Eisenberger et al., 1986). A meta-analysis study finds that organizational support positively links to task performance (Rhoades and Eisenberger, 2002), and a longitudinal study further reveals that organizational support positively predicts task performance in 1 year (Affuso et al., 2023). Besides, this potential influence might be stronger for Chinese employees. Traditionally, Chinese employees are supposed to view their organization as a symbolic family, especially in some organizations such as state-owned enterprises (Zhang et al., 2012), which might increase the potential influence of organizational support for Chinese employees. Empirical studies also support this notion. As revealed in a meta-analysis, organizational support is more strongly related to job attitudes and performance in the East than in the West (Rockstuhl et al., 2020). Hence, it is of greater significance to investigate the potential influence of organizational support on task performance in Chinese context. Although some studies have tested the direct relationship between organizational support and task performance (Rhoades and Eisenberger, 2002), previous studies have not adequately explored the underlying mechanisms. Based on social exchange theory, self-determination theory, happy-productive worker hypothesis, and goal setting theory, this study constructed and empirically tested a theoretical model to investigate the mediating mechanisms and to provide scientific suggestions for developing a multi-stage strategy to enhance Chinese employees’ task performance.

### 1.1 Organizational support and task performance

Organizational support can be a potential cause for task performance. According to social exchange theory (SET), social behaviors can be the result of exchanges guided by the principle of reciprocity. When one party provides material and non-material support, the counterpart is inclined to exhibit more favorable behaviors as a form of reciprocation (Homans, 1958). Specifically, when an organization cares about the well-being and interests of employees, they tend to reciprocate with higher levels of performance (Wayne et al., 1997). Empirical findings repeatedly support this view. A meta-analysis study indicates a positive correlation between organizational support and task performance (Rhoades and Eisenberger, 2002). The correlation is stronger in Eastern countries, as revealed by a cross-culture meta-analysis (Rockstuhl et al., 2020). A cross-lagged study further finds that organizational support positively predicts employees’ proactive behaviors toward the organization 4 months later, while employees’ proactive behaviors cannot predict organizational support (Caesens et al., 2015). In addition, a longitudinal study shows that teacher support can positively predict students’ academic performance in 1 year (Affuso et al., 2023). Moreover, an experimental study further confirms that organizational social recognition enhances both individual and team performance (Li et al., 2016). Based on the above analyses, this study hypothesized:

*H1*: Organizational support positively predicts employees’ task performance.

### 1.2 The mediating role of job satisfaction

Job satisfaction can play a mediating role between organizational support and task performance. Essentially, job satisfaction is defined as an evaluation of the favorability of a job, continuously ranging from positive to negative (Weiss, 2002), and includes internal satisfaction and external satisfaction (Weiss et al., 1967).

Job satisfaction can be a potential consequence of organizational support. Self-determination theory suggests that social support satisfies relational needs, and boosts well-being (Deci and Ryan, 1985). Besides, organizational support increases overall job satisfaction by meeting social emotional needs, improving performance reward expectations, and expressing the availability of assistance when needed as well (Rhoades and Eisenberger, 2002). In line with this notion, a meta-analysis confirms that organizational support is positively related to job satisfaction (Rhoades and Eisenberger, 2002). Moreover, a longitudinal study reveals that employees’ supervisor support can positively predict their job satisfaction in 2 years (Jokisaari and Nurmi, 2009).

Meanwhile, employees’ job satisfaction can be an underlying reason for their task performance. According to the happy-productive worker hypothesis, employees who are more satisfied with their jobs will better perform the tasks (Zelenski et al., 2008). Job satisfaction is one of the reflections on happiness (Cropanzano and Wright, 2001). The happy individuals also incline to solve problems creatively and fulfill work responsibilities actively, thus leading to better performance in their work (Fredrickson and Branigan, 2001; Zelenski et al., 2008; Wan et al., 2022). Several meta-analysis studies confirm that job satisfaction is positively related to task performance (Petty et al., 1984; Riketta, 2008; Whitman et al., 2010). Furthermore, some longitudinal studies reveal that task performance can be predicted by positive moods 3 weeks earlier (Tsai et al., 2007) and by job satisfaction 1 year before (Alessandri et al., 2017). Moreover, an experimental study further confirms that happiness facilitates performance (Oswald et al., 2015). In consideration that organizational support positively predicts job satisfaction, which further positively predicts task performance, a second hypothesis can be put forward:

*H2*: Job satisfaction mediates the association between organizational support and task performance.

### 1.3 The mediating role of organizational commitment

Organizational commitment can also play a mediating role between organizational support and task performance. Organizational commitment refers to employees’ tendency to serve their organizations on an ongoing basis (Becker, 1960). It encompasses a heightened sense of unilateral commitment to the organization, which is reflected through the allocation of personal resources to the organization and the acquisition of skills that can only be used in the current organization (Becker, 1960). It can be understood as the degree of employees’ identification with and intensity of involvement in the organization, suggesting that high organizational commitment is characterized by three features: a strong belief in the goals and values of the organization, a clear willingness to contribute to the organization, and a strong desire to maintain membership in the organization (Mowday et al., 1979).

Organizational support might influence organizational commitment of the employee. According to social exchange theory, the social behavior can be seen as an exchange process with both parties following the principle of reciprocity (Homans, 1958). When an organization cares for the well-being of its members and looks after their interests, the employees not only have positive attitudes toward the organization but also tend to develop a sense of obligation to better serve the organization (Cropanzano et al., 2001). Empirical studies also support this view. A meta-analysis study based on 588 studies indicates that organizational support is positively related to affective organizational commitment (Kurtessis et al., 2017). Furthermore, a cross-lagged study finds that organizational support can predict organizational commitment in 2 and 3 years, while organizational commitment cannot significantly predict organizational support later (Rhoades et al., 2001). Moreover, an experimental study further confirms that improved organizational climate, which includes organizational support (Sun et al., 2008), can enhance employees’ organizational commitment (Gattiker et al., 2014).

Meanwhile, higher organizational commitment might lead to better task performance. According to goal setting theory, actions are governed by conscious intention, which can be manifested as goals (Locke, 1968). Organizational goals that accepted by employees contribute to greater individual effort and persistence, which further enhances performance (Vigoda-Gadot and Angert, 2007). Employees with high organizational commitment not only hold a firmer belief in and evinced stronger desire for the realization of the organizational goals (Mowday et al., 1979), but also tend to set higher performance goals themselves along with more effort to realized them (Wallace, 1995). Thus, they exhibit better performance in general. Empirical studies also support this view. For instance, a meta-analysis study confirms that organizational commitment is positively related to task performance (Wang et al., 2019). Furthermore, a longitudinal study reveals that organizational commitment positively predicts job performance in 3 years (Maia et al., 2016). Moreover, a meta-analysis of panel studies finds that organizational commitment can positively predict task performance at both short-time and long-time lags, while performance cannot significantly predict organizational commitment (Riketta, 2008). For the reason that organizational support positively predicts organizational commitment and organizational commitment positively predicts task performance, the third hypothesis could be posited:

*H3*: Organizational commitment mediates the association between organizational support and task performance.

### 1.4 The multiple mediation model

Job satisfaction might lead to organizational commitment. According to social exchange theory, employees satisfied with their job are inclined to have solid willingness to contribute to the organization (Cropanzano et al., 2001). They usually have greater recognition and attachment to work, along with increased job involvement, which further contributes to stronger organizational commitment (Culibrk et al., 2018). Empirical studies also support this view. A meta-analysis research confirms that job satisfaction is positively related to organizational commitment (Fragkos et al., 2020), and a longitudinal study further reveals that job satisfaction can positively predict organizational commitment in 6 months (Abdelmoteleb, 2019). For the reason that both job satisfaction and organization commitment mediate between organizational support and task performance, while job satisfaction positively predicts organizational commitment, this study hypothesized:

*H4*: Job satisfaction and organizational commitment play serial mediating role in the relationship between organizational support and task performance.

### 1.5 The present study

**Figure 1:**
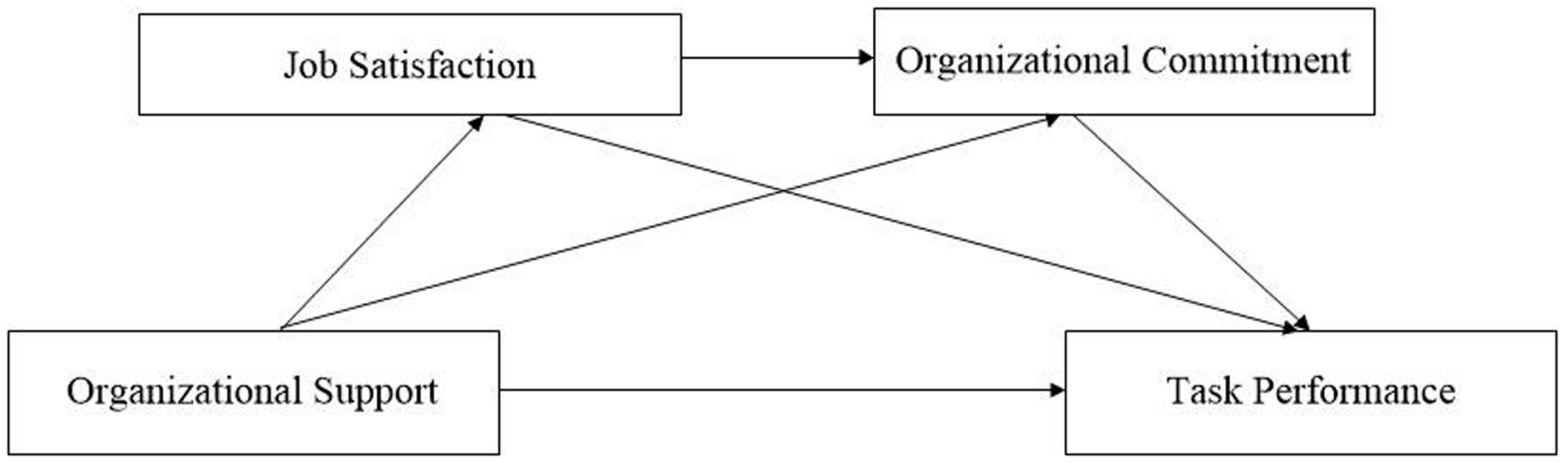
The proposed multiple mediation model.

To uncover the underlying relationship between organizational support and task performance, this study took a process-oriented approach by testing a serial mediation model (see Figure 1). In particular, the following questions were addressed: (a) whether organizational support positively associates with task performance through (b) job satisfaction and (c) organizational commitment; and (d) whether job satisfaction and organizational commitment play a serial mediating role in the association.

## 2 Method

### 2.1 Participants

Convenient sampling was applied in this study. The minimal sample size was determined through calculations using the G*Power 3.1.9.6 computer program software with the effect sizes between variables obtained from reports of previous meta-analysis studies of the relevant concepts (Lee et al., 2000; Rhoades and Eisenberger, 2002; Ng et al., 2009; Rockstuhl et al., 2020). Power analysis indicated the minimal sample size for this study is 329 to keep type I error less than 0.05 and power higher than 0.95. After obtaining informed consent, an anonymous self-report questionnaire was distributed to a cluster sample of 720 participants from 12 audit firms in Beijing in December 2020. Participants consisted of 367 males (50.97%) and 353 females (49.03%). The age of participants ranged from 20 to 68 years old (*M* = 32.43, *SD* = 8.63). The demographic characteristics of the participants are presented in Table 1.

**Table 1:** Demographic characteristics of the sample.

Variables		Frequency	Percentage
Gender	Male	367	50.97%
	Female	353	49.03%
Education level	Vocational college diploma or below	285	39.58%
	Bachelor	405	56.25%
	Master or Doctor	30	4.17%
Annual income	CNY 60,000 or below	201	27.92%
	(CNY 60,000, CNY 120,000)	295	40.97%
	(CNY 120,000, CNY 180,000)	129	17.92%
	(CNY 180,000, CNY 240,000)	52	7.22%
	Higher than CNY 240,000	43	5.97%
Marital status	Single	308	42.78%
	Married	384	53.33%
	Divorced	17	2.36%
	Others	11	1.53%
Work seniority	5 years or less	265	36.81%
	(5 years, 10 years)	210	29.17%
	(10 years, 15 years)	112	15.56%
	More than 15 years	133	18.47%

### 2.2 Measures

Organizational support was assessed by Perceived Organizational Support Scale. This scale was developed by Eisenberger et al. (2001) and has been used among the Chinese participants (e.g., Wang et al., 2017). It uses a five-point Likert scale ranging from 1 (strongly disagree) to 5 (strongly agree) and consists of 6 items. Responses of all 6 items were averaged, with higher scores indicating higher levels of organizational support. The scale was reliable with the Cronbach’s alpha coefficient of 0.94 and the first extracted factor explaining 76.16% of the total variance. CFA supported the structural validity of the scale was acceptable with SRMR = 0.01, CFI = 0.99, TLI = 0.99. Task Performance Subscale of the Job Performance Scale (Han et al., 2007) was used to measure task performance. The scale consists of 10 items. Participants answered each item on a five-point Likert scale ranging from 1 (never) to 5 (always). The mean score was calculated, with higher scores indicating higher levels of task performance. Cronbach’s alpha coefficient in the present study was 0.76, with the first extracted factor explaining 43.27% of the total variance. The job satisfaction was assessed by Chinese version of Minnesota Satisfaction Short-Form Questionnaire (which was publicly provided by Department of Psychology, University of Minnesota, with the following link: https://vpr.psych.umn.edu/sites/vpr.umn.edu/files/files/chinese_msq_1977_short_form_2.pdf), which was developed by Weiss et al. (1967). The scale consists of 20 items and uses a five-point Likert scale ranging from 1 (strongly dissatisfied) to 5 (strongly satisfied). Responses of all 20 items were averaged, with higher scores indicating higher levels of job satisfaction. The Cronbach’s alpha coefficient in this study was 0.96, with the first extracted factor explaining 56.25% of the total variance. CFA supported the structural validity of the scale was acceptable with SRMR = 0.04, CFI = 0.88, TLI = 0.87. Organizational Commitment Short-Form Questionnaire, developed by Mowday et al. (1979) and revised by Long (2002), was used to measure organizational commitment. It consists of 9 items and uses a four-point Likert scale ranging from 1 (strongly disagree) to 4 (strongly agree). Responses of all 9 items were averaged, with higher scores indicating higher levels of organizational commitment. The Cronbach’s alpha coefficient in the current study was 0.94, with the first extracted factor explaining 68.04% of the total variance. CFA supported the structural validity of the scale was acceptable with SRMR = 0.03, CFI = 0.95, TLI = 0.94.

### 2.3 Statistical analyses

The data analysis was conducted with SPSS 26.0. Firstly, potential common method bias was checked. After calculations of bivariate correlations, Hayes’ PROCESS macro Model 4 (mediation model, i.e., X → M → Y) was performed to test the mediating effect of job satisfaction and organizational commitment, respectively. Then, Model 6 (multiple mediation model, i.e., X → M1 → M2 → Y) was performed to test the hypothesized serial mediating effect of job satisfaction and organizational commitment in the relationship between organizational support and task performance (Hayes, 2013). Before application of Model 4 and 6, standardized scores of all variables were computed. The significance of indirect effects was tested with bias-corrected percentile bootstrap method (with 5,000 resamples).

## 3 Results

### 3.1 Descriptive statistics and correlation analysis

Descriptive statistics and correlation matrix of organizational support, work autonomy, job satisfaction, and organizational commitment are provided in Table 2. Results of bivariate correlation analysis show that organizational support, job satisfaction, organizational commitment, and task performance were significantly and positively correlated with each other (*p* < 0.001), supporting Hypothesis 1.

**Table 2:** Descriptive statistics and correlation matrix of all variables.

Variables	*M*	*SD*	1	2	3	4
OS	3.54	0.86	1					
JS	3.88	0.65	0.62^***^	1			
OC	3.09	0.59	0.68^***^	0.61^***^	1	
TP	3.95	0.59	0.30^***^	0.33^***^	0.38^***^	1

OS, Organizational Support; JS, Job Satisfaction; OC, Organizational Commitment; TP, Task Performance. N = 720, ^***^p < 0.001, same below.

### 3.2 Mediating effect of job satisfaction

The PROCESS macro for SPSS (Model 4, i.e., X → M → Y) was applied to test Hypothesis 2. The results indicated that task performance was positively predicted by organizational support (*b* = 0.30, *p* < 0.001, Model 1 of Table 3), which also supported Hypothesis 1. Moreover, organizational support positively predicted job satisfaction (*b* = 0.62, *p* < 0.001, Model 2 of Table 3), which in turn predicted task performance (*b* = 0.22, *p* < 0.001, Model 3 of Table 3). Job satisfaction therefore played a mediating role in the link between organizational support and task performance (indirect effect = 0.14, 95% CI = [0.07–0.22]), supporting Hypothesis 2.

**Table 3:** Testing the mediating effect of job satisfaction and organizational commitment respectively.

Regression model	Outcome	Predictors	*R*	*R^2^*	*F*	*b*	*SE*	*t*
Model 1	TP		0.30	0.09	72.73^***^			
		OS				0.30	0.04	8.53^***^
Model 2	JS		0.62	0.38	443.94^***^			
		OS				0.62	0.03	21.07^***^
Model 3	TP		0.35	0.12	50.21^***^			
		OS				0.17	0.04	3.71^***^
		JS				0.22	0.04	5.02^***^
Model 4	OC		0.68	0.46	613.81^***^			
		OS				0.68	0.03	24.78^***^
Model 5	TP		0.38	0.15	62.18^***^			
		OS				0.08	0.05	1.81
		OC				0.32	0.05	6.85^***^

Each variable was standardized before brought into the regression equation, same below. ***p < 0.001.

### 3.3 Mediating effect of organizational commitment

Hypothesis 3 was also tested with the PROCESS macro for SPSS (Model 4, i.e., X → M → Y). Results indicated that organizational support positively predicted organizational commitment (*b* = 0.68, *p* < 0.001, Model 4 of Table 3), which further positively predicted task performance (*b* = 0.32, *p* < 0.001, Model 5 of Table 3). Organizational commitment therefore played a mediating role in the link between organizational support and task performance (indirect effect = 0.22, 95% CI = [0.16–0.28]), supporting Hypothesis 3.

### 3.4 Serial mediating effects of job satisfaction and organizational commitment

The PROCESS macro for SPSS (Model 6, i.e., X → M1 → M2 → Y) was used to test the multiple mediation model. Results showed that organizational support positively predicted both job satisfaction (*b* = 0.62, *p* < 0.001, Model 2 of Table 4) and organizational commitment (*b* = 0.49, *p* < 0.001, Model 3 of Table 4); Furthermore, job satisfaction positively predicted organizational commitment (*b* = 0.31, *p* < 0.001, Model 3 of Table 4); Besides, both job satisfaction (*b* = 0.14, *p* < 0.01, Model 4 of Table 4) and organizational commitment (*b* = 0.27, *p* < 0.001, Model 4 of Table 4) positively predicted task performance. The pathway of “organizational support → job satisfaction → organizational commitment → task performance” was significant (indirect effect =0.05, 95% CI = [0.03–0.08]), which supported Hypothesis 4 (Table 5).

**Table 4:** Testing the serial mediating effects of job satisfaction and organizational commitment.

Regression Model	Outcome	Predictors	*R*	*R^2^*	*F*	*b*	*SE*	*t*
Model 1	TP		0.30	0.09	72.73^***^					
		OS						0.30	0.04	8.53^***^
Model 2	JS		0.62	0.38	443.94^***^					
		OS						0.62	0.03	21.07^***^
Model 3	OC		0.72	0.52	387.52^***^					
		OS						0.49	0.03	14.84^***^
		JS							0.31	0.03	9.35^***^
Model 4	TP		0.40	0.16	44.97^***^					
		OS						0.03	0.05	0.64
						JS				0.14	0.05	3.02^**^
						OC				0.27	0.05	5.51^***^

^**^p < 0.01.

**Table 5:** Testing the path ways of the multiple mediation model.

Model pathways	Estimated	*SE*	Boot LLCI	Boot ULCI
OS→JS → TP	0.09	0.04	0.02	0.17
OS→OC → TP	0.13	0.03	0.07	0.19
OS→JS → OC → TP	0.05	0.01	0.03	0.08
Total indirect effect	0.27	0.04	0.20	0.35

## 4 Discussion

This study investigated the relationship between organizational support and task performance with mediating roles of job satisfaction and organizational commitment. The results indicated that organizational support can be indirectly linked to task performance through both job satisfaction and organizational commitment respectively, as well as via the serial multiple mediation of job satisfaction and organizational commitment. The results supported our theoretical hypotheses and extended the findings of previous studies by providing potential mechanisms linking organizational support to employees’ task performance.

### 4.1 Organizational support and task performance

Correlation analysis indicated that organizational support was positively linked to task performance, which supported Hypothesis 1. The finding is congruent with the view of social exchange theory (Homans, 1958) as well as previous empirical findings (Rhoades and Eisenberger, 2002; Caesens et al., 2015; Li et al., 2016; Rockstuhl et al., 2020). The positive relationship indicated that employees’ excellent performance could be in return for the value organization place on their contributions and the concern for their well-being.

### 4.2 Mediating roles of job satisfaction and organizational commitment

Job satisfaction and organizational commitment both played mediating roles, respectively, in the association between organizational support and task performance. For the mediating role of job satisfaction, empirical results from this study confirmed Hypothesis 2, which was deduced from self-determination theory (Deci and Ryan, 1985) and happy-productive worker hypothesis (Zelenski et al., 2008). Organizational support can meet the basic needs of the employee, facilitating satisfaction with job (Deci and Ryan, 1985). The satisfied individuals incline to reward the organization with more favorable behaviors (Homans, 1958; Eisenberger et al., 1986), to solve problems creatively, and to fulfill work responsibilities actively, thus leading to better performance (Fredrickson and Branigan, 2001; Zelenski et al., 2008; Wan et al., 2022). Similarly, for the mediating role of organizational commitment, empirical result from this study verified Hypothesis 3, which was proposed derived from social exchange theory (Homans, 1958) and goal setting theory (Locke, 1968). It is easier for the employees, whose well-being was cared by the organization, to develop a sense of obligation to better serve the organization (Cropanzano et al., 2001). They usually have a strong belief and desire for the realization of the goal of the organization (Mowday et al., 1979), which contributes to high levels of performance (Vigoda-Gadot and Angert, 2007). Consequently, both employees’ satisfaction with the job and commitment to the organization play important roles for bridging between organizational support and task performance of employee. Although it is worth for the organization providing abundant social support for employees, the support organization could provide is not unlimited in consideration of the cost as well as capacity. Hence, job satisfaction and organizational commitment of the employee should also be paid close attention.

### 4.3 Serial mediating effects of job satisfaction and organizational commitment

Findings regarding the serial mediating effect verified “organizational support → job satisfaction → organizational commitment → task performance” pathway, which supported Hypothesis 4 that proposed mainly based on social exchange theory (Cropanzano et al., 2001). It is also congruent with the findings of previous empirical studies demonstrating relationship between each variable dyad. Specially, organizational support positively predicts job satisfaction (Dreer, 2022; Walsh and Kabat-Farr, 2022; Patterer et al., 2023), which further positively predicts organizational commitment (Fragkos et al., 2020; Marta et al., 2021; Thi et al., 2021), that can finally positively predict task performance (Che et al., 2021; Fragoso et al., 2022; Lee et al., 2022). In other words, individuals with high organizational support tend to be satisfied with their job, which increases their organizational commitment, that directly improves their task performance. For relationship between job satisfaction and organizational commitment, employees higher in job satisfaction usually recognize and attach stronger to the work, have deeper job involvement (Culibrk et al., 2018), want to contribute more to the organization (Cropanzano et al., 2001), which all facilitate organizational commitment (Culibrk et al., 2018). The finding of the serial chain mediating effect reveals the internal mediating mechanism of how organizational support links to task performance, which extends the theories and deepens the understanding for the relationship. Providing sufficient organizational support to employees can foster their job satisfaction directly, enhance their organizational commitment thereafter, and finally promote their task performance.

### 4.4 Implication

The findings significantly advance our comprehension of the potential mechanisms linking organizational support to task performance. Moreover, they offer valuable scientific insights to propose a multi-stage strategy to enhance employees’ task performance.

Organizational support was found positively linked to task performance. In other words, when the well-being and interests of employees are cared by the organization, they are inclined to respond with higher levels of performance (Wayne et al., 1997). These findings suggest that employees’ outstanding performance may be in return for the value the organization assigns on their contributions and the concern for their well-being. Consequently, organizations should be generous with social support to their employees, which is worthwhile in the long run as it contributes to improvement of employees’ performance and can finally enhance realization of organizational goals (Tett and Burnett, 2003).

Although providing organizational support might be an effective way enhancing employees’ task performance, it is not unlimited or without cost. As revealed in the previous section, job satisfaction and organizational commitment played a serial mediating role in the association between organizational support and task performance. In other words, providing substantial organizational support to employees may firstly foster their job satisfaction, subsequently enhance their organizational commitment, and ultimately promote their task performance, which implies a potential multi-stage strategy to enhance employees’ task performance. In addition to organizational support, any practices that can boost job satisfaction or advance organizational commitment should be adopted, such as implementing a fair performance evaluation system and providing clear organizational objectives (Kim, 2023), developing workplace safety policies (Bajrami et al., 2021), and implementing effective participation and communication mechanisms (Lee et al., 2021).

To sum up, we recommend a strategic focus on improving organizational support, while also paying attention to the serial multiple mediating role of job satisfaction and organizational commitment to effectively improve employees’ task performance.

### 4.5 Limitations and future directions

The current study has provided a theory on how organizational support links to task performance. However, several limitations still exist and need to be addressed in future studies. Firstly, although it is reasonable to deduce an “organizational support → job satisfaction → organizational commitment → task performance” path from theories and previous empirical studies, causal relationships should be taken with caution since questionnaire survey was the method for data collection. In future research, experimental or intervention methods should be adopted to make stronger claims on the causal direction of the effects. Secondly, more work is needed to fully uncover the mechanisms between organizational support and task performance. Although the paths revealed in this study are important, there may be other paths linking organizational support to organizational commitment. Future research should continue to explore possible mediating and moderating variables between organizational support and organizational commitment. Thirdly, future study should use a random sample to further validate the findings of this study. Despite these limitations, current study has advanced our understanding of the mechanisms linking organizational support to organizational commitment. The findings may help provide practical advices for improving task performance.

## 5 Conclusion

Despite existing evidence of the general connection between organizational support and task performance, exploring the underlying mechanisms of their relationship is crucial for developing a multi-stage strategy to enhance employees’ task performance. This study investigated the relationship between organizational support and task performance with a serial multiple mediating role of job satisfaction and organizational commitment. Specially, the following results were revealed in this study:

1. Organizational support was positively associated with employees’ job satisfaction, organizational commitment, and task performance. Job satisfaction and organizational commitment were both positively associated with task performance. Job satisfaction was also positively associated with organizational commitment.
2. Organizational support indirectly linked to task performance through the mediating roles of job satisfaction and organizational commitment, respectively. Organizational support can meet employees’ basic needs, promoting job satisfaction or elevating levels of organizational commitment, thus indirectly linked to employees’ task performance.
3. Organizational support was indirectly associated with task performance through the chain mediating role of job satisfaction and organizational commitment. Individuals with high organizational support often find satisfaction in their work, which further increases their organizational commitment and finally enhances their task performance.

## Data availability statement

The raw data supporting the conclusions of this article will be made available by the authors, without undue reservation.

## Ethics statement

The studies involving humans were approved by Human Research Committee of Beijing University of Posts and Telecommunications. The studies were conducted in accordance with the local legislation and institutional requirements. The participants provided their written informed consent to participate in this study.

## Author contributions

XC: Conceptualization, Data curation, Methodology, Writing – original draft, Writing – review & editing. JY: Conceptualization, Data curation. Methodology, Writing – original draft, Writing – review & editing. AL: Methodology, Writing – original draft, Writing – review & editing. WZ: Writing – original draft, Writing – review & editing. XW: Writing – review & editing. PW: Writing – review & editing. JW: Writing – original draft, Writing – review & editing.
